# Transcriptomic Profiling Unravels the Molecular Mechanisms of *GmCML*-Mediated Resistance to *Fusarium oxysporum* in Soybean

**DOI:** 10.3390/plants14203222

**Published:** 2025-10-20

**Authors:** Runnan Zhou, Jia You, Jinrong Li, Xue Qu, Yuxin Shang, Honglei Ren, Jiajun Wang

**Affiliations:** Soybean Research Institute, Heilongjiang Academy of Agricultural Sciences, Harbin 150086, China; runnanzhou@126.com (R.Z.); rokiyou@126.com (J.Y.); haasljr@163.com (J.L.); hlj_quxue1082@126.com (X.Q.); syx17804588278@126.com (Y.S.)

**Keywords:** soybean, *Fusarium oxysporum*, transcriptome, *GmCML*, gene function characterization, haplotype analysis

## Abstract

*Fusarium oxysporum*-induced root rot severely threatens global soybean production, yet limited understanding of resistance mechanisms constrains breeding progress. This study conducted comparative transcriptomic analysis between highly resistant (Xiaoheiqi) and susceptible (L83-4752) soybean accessions following pathogen inoculation across four time points (8–17 days post-infection). RNA-seq analysis identified 1496 differentially expressed genes following pathogen challenge. KEGG pathway enrichment analysis revealed significant enrichment in MAPK signaling pathway (12 genes) and plant–pathogen interaction pathway (13 genes). Eight genes co-occurred in both pathways, with *GmCML* (*Glyma.10G178400*) exhibiting the most dramatic differential expression among these candidates. This gene encodes a 151-amino acid calmodulin-like protein showing 185-fold higher expression in resistant plants at 17 days post-inoculation, confirmed by qRT-PCR validation. Functional validation through transgenic hairy root overexpression demonstrated that *GmCML* significantly enhanced disease resistance by coordinately activating antioxidant defense systems. Overexpression of *GmCML* in transgenic soybean enhanced resistance to *F. oxysporum* by modulating the activity of antioxidant enzymes (superoxide dismutase, SOD; peroxidase, POD; catalase, CAT) and the accumulation of osmoregulatory substances (proline and soluble sugars). Population genetic analysis of 295 diverse soybean accessions revealed three *GmCML* haplotypes based on promoter region polymorphisms. Two favorable variants (Hap2 and Hap3) conferred significantly lower disease indices and exhibited evidence of positive selection during domestication, indicating evolutionary importance in disease resistance. This research provides the first comprehensive characterization of *GmCML*’s role in soybean–*Fusarium* interactions, establishing this calmodulin-like protein as a regulatory hub linking calcium signaling to coordinated defense responses. The identified natural variants and functional mechanisms offer validated targets for both marker-assisted breeding and genetic engineering approaches to enhance soybean disease resistance.

## 1. Introduction

Soybean (*Glycine max* (L.) Merr.) ranks among the world’s most economically important crops, providing essential plant protein and vegetable oil for human consumption and animal feed [1]. With annual global production exceeding 350 million metric tons across six continents [2,3], soybean cultivation faces mounting challenges from biotic stresses, particularly soil-borne fungal diseases that reduce yields by 10–60% depending on environmental conditions and pathogen pressure [4,5].

Fusarium root rot (FRR) represents one of the most destructive soil-borne diseases affecting soybean worldwide, causing substantial yield losses and economic damage [6]. In North America, FRR reduces yields by 15–30% in severely affected fields, with annual losses exceeding $100 million in the United States alone. Major soybean-producing states including Iowa, Illinois, Indiana, and Minnesota report widespread FRR occurrence, with disease incidence reaching 40–70% in some fields under favorable conditions for pathogen development [7]. In South America, Brazil and Argentina—which together account for approximately 50% of global soybean production—face increasing FRR pressure as cultivation intensifies [8].

In China, which produces approximately 17 million metric tons of soybeans annually (representing about 8% of global production), FRR has emerged as a critical concern threatening domestic food security and agricultural sustainability [9,10]. The disease is particularly severe in the northeastern provinces of Heilongjiang, Jilin, and Liaoning, which comprise the primary soybean production belt, representing over 40% of China’s cultivation area [10]. Recent systematic surveys conducted between 2018 and 2022 across these provinces documented alarming FRR incidence rates: Heilongjiang Province showed 35–60% incidence in monitored fields, Jilin Province exhibited 25–50% incidence, and Liaoning Province recorded 30–55% incidence [10,11]. Disease severity has intensified markedly under continuous soybean cropping systems, which have become increasingly common due to agricultural intensification and limited crop rotation options [12]. Economic analyses estimate that FRR causes annual losses of approximately 2–3 million metric tons in northeastern China, valued at over $800 million USD based on current commodity prices [13]. Beyond quantitative yield reduction, FRR severely compromises seed quality through decreased germination rates (often reduced by 15–30% in moderately infected seed lots), diminished protein content (reductions of 2–5 percentage points), reduced oil content (decreases of 1–3 percentage points), and mycotoxin contamination, which further diminishes the marketability and economic value of harvested crops [14].

A complex of *Fusarium* species causes FRR, with *F. solani*, *F. acuminatum*, *F. graminearum*, and *F. oxysporum* most reported as causal agents [15]. While *F. solani* has historically been considered the predominant pathogen in many regions, recent comprehensive pathogen surveys reveal considerable variation in species composition depending on geographic location, soil conditions, environmental factors, and cropping history [16]. In northeastern China, where our research is focused, systematic field surveys and pathogen isolation studies conducted over three growing seasons (2019–2021) have identified *F. oxysporum* as a significant and increasingly prevalent causal agent of soybean root rot, particularly in fields with extended soybean monoculture (≥3 consecutive years) [11]. Isolation frequency data from 450 diseased plant samples collected across 75 fields in Heilongjiang Province revealed that *F. oxysporum* comprised 38% of recovered isolates, followed by *F. solani* (32%), *F. graminearum* (18%), and *F. acuminatum* (12%). Pathogenicity assays demonstrated that *F. oxysporum* isolates from this region exhibited virulence levels comparable to or exceeding those of *F. solani* isolates when inoculated onto susceptible soybean cultivars under controlled conditions [17]. Unlike the vascular wilt syndrome caused by formae speciales of *F. oxysporum* in other crops (such as *F. oxysporum f.* sp. *lycopersici* in tomato), the isolates recovered from diseased soybean roots in our region cause typical root rot symptoms including cortical browning, extensive necrosis, and severely reduced root system development without characteristic vascular discoloration [18].

These pathogens exhibit remarkable soil persistence, surviving as chlamydospores for extended periods (often 5–10 years in the absence of host plants) and establishing infection through root wounds, natural openings, or direct penetration of root tissues [19]. Following initial colonization, they invade vascular tissues, disrupt water and nutrient transport through vessel occlusion and toxin production, and produce characteristic disease symptoms of root necrosis, vascular browning, foliar chlorosis, stunting, and ultimately plant death [20]. The disease proves particularly problematic in continuous cropping systems and poorly drained soils, where pathogen inoculum densities can reach economically damaging levels exceeding 10^3^–10^4^ colony-forming units per gram of soil [4].

Current FRR management relies heavily on chemical fungicides, crop rotation, and cultural practices [21,22]. However, these approaches face significant limitations: environmental concerns associated with fungicide applications, rapid development of fungicide resistance in pathogen populations, substantial economic constraints for smallholder farmers (fungicide costs often exceeding $50–80 per hectare), and inconsistent efficacy across diverse production environments and pathogen species complexes [23,24]. Consequently, developing genetically resistant cultivars represents the most economically viable, environmentally sustainable, and broadly applicable long-term solution for disease management [25,26]. Plant immunity involves complex signaling networks that perceive pathogen-associated molecular patterns (PAMPs) through pattern recognition receptors (PRRs) and initiate appropriate defense responses [27,28,29]. In soybean–*Fusarium* interactions, defense mechanisms encompass PRR activation, mitogen-activated protein kinase (MAPK) cascade signaling, reactive oxygen species (ROS) generation, and downstream effector responses including pathogenesis-related protein expression, phytoalexin biosynthesis, and cell wall reinforcement [30,31]. Calcium (Ca^2+^) signaling plays a central role in coordinating these defense networks, with spatially and temporally defined Ca^2+^ concentration fluctuations serving as critical secondary messengers that activate downstream defense cascades [32,33,34]. Calmodulin-like (CML) proteins, a plant-specific family of calcium sensors, decode these Ca^2+^ signatures and translate them into appropriate cellular responses during development, stress adaptation, and pathogen defense [33,34]. Unlike highly conserved calmodulin proteins found across eukaryotes, CML proteins exhibit substantial structural diversity with variable numbers of EF-hand calcium-binding domains (typically 2–6 EF-hands per protein) and display specialized functional roles in diverse signaling pathways [35]. Recent studies in model plants demonstrate that several CML proteins function as positive or negative regulators of disease resistance, with AtCML24, AtCML9, and AtCML43 in *Arabidopsis* showing direct involvement in modulating resistance against bacterial pathogens through regulation of defense gene expression and immune receptor function [36,37].

In soybean, genome-wide analyses have identified the *CML* gene family and began elucidating their functional roles in stress responses. Zeng et al. [38] performed comprehensive identification and characterization of 57 EF-hand proteins in the soybean genome, including multiple CML family members, and analyzed their phylogenetic relationships, gene structures, chromosomal distributions, and expression patterns under various stress conditions. Their analysis revealed that many soybean EF-hand proteins, including CMLs, showed differential expressions in response to environmental stresses such as salt, drought, and nutrient deficiency, suggesting their involvement in stress signaling pathways. The authors noted that the expansion and diversification of the EF-hand protein family in soybean likely reflects the complex regulatory networks required for adaptation to diverse environmental challenges. Yadav et al. [39] conducted an in-depth investigation specifically focused on calmodulin-like proteins in soybean, identifying 50 *GmCML* genes distributed across 18 chromosomes. Their comprehensive analysis included phylogenetic classification, gene structure analysis, chromosomal localization, identification of conserved motifs and cis-regulatory elements, and tissue-specific expression profiling. Importantly, they examined the transcriptional responses of *GmCML* genes to biotic stress, specifically insect herbivory by *Spodoptera litura*, and found that several *GmCML* genes were significantly upregulated following insect attack, with some showing tissue-specific induction patterns. Their promoter analysis revealed enrichment of defense-responsive cis-elements including W-boxes, MYB binding sites, and stress-responsive elements in multiple *GmCML* genes. The authors concluded that *GmCML* genes play important roles in calcium-mediated signaling during plant defense responses against insect pests. However, despite these important genomic and expression studies, the specific functions of individual *GmCML* genes in disease resistance, particularly against fungal pathogens, remained largely unexplored. While Yadav et al. [39] demonstrated the involvement of *GmCML* genes in insect defense, no functional validation had been performed for any *GmCML* gene in response to pathogen challenge, and the downstream mechanisms by which specific CML proteins might contribute to disease resistance were unknown.

RNA sequencing (RNA-seq) technology has revolutionized plant pathology research by enabling comprehensive transcriptomic profiling of host–pathogen interactions at unprecedented resolution [40]. This high-throughput approach facilitates systematic identification of defense-responsive genes, characterization of temporal expression dynamics, and discovery of novel resistance mechanisms [41]. Several recent studies have successfully employed RNA-seq to dissect soybean responses to various pathogens, revealing complex gene regulatory networks and identifying candidate resistance genes for functional validation [42].

In this study, we conducted a comprehensive investigation to elucidate the molecular mechanisms underlying soybean resistance to *F. oxysporum* root rot. Through comparative transcriptomic analysis of contrasting genotypes, we identified a calmodulin-like gene, *GmCML*, as a key regulator of disease resistance. Functional validation through transgenic approaches, combined with population genetic analysis, revealed that *GmCML* enhances resistance by coordinating antioxidant defense systems and osmoregulatory responses. These findings advance our understanding of calcium signaling in plant immunity and provide validated targets for improving soybean disease resistance through both conventional breeding and biotechnological approaches.

## 2. Results

### 2.1. Contrasting Disease Phenotypes and Physiological Responses Between Resistant and Susceptible Soybean Accessions

**Figure 1:**
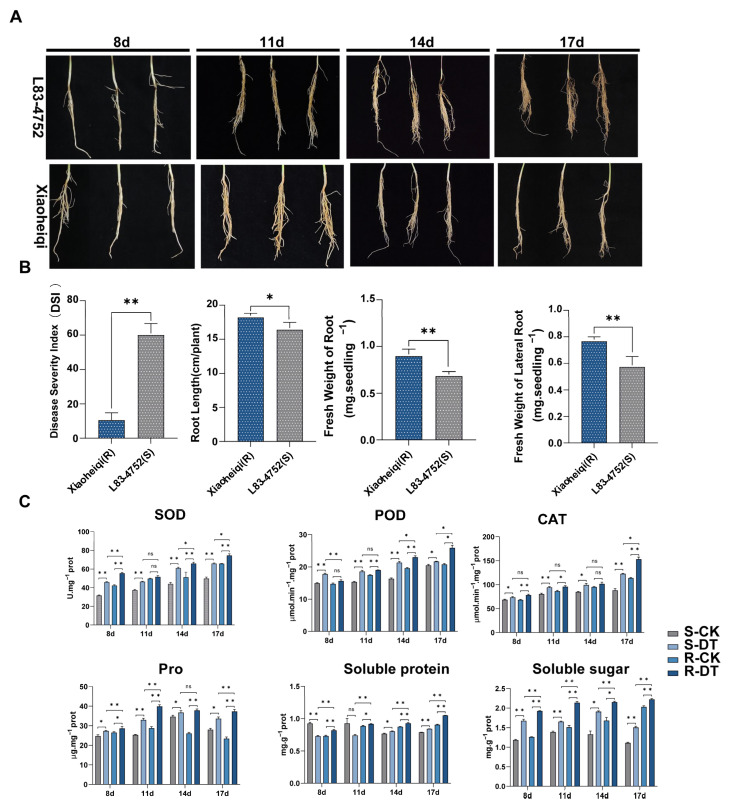
**Differential resistance to Fusarium oxysporum and associated physiological changes in contrasting soybean genotypes.** (**A**) Representative root systems of resistant accession Xiaoheiqi (upper panels) and susceptible accession L83-4752 (lower panels) at 8, 11, 14, and 17 days post-inoculation with *F. oxysporum*, showing progressive disease development. Scale bar = 2 cm. (**B**) Quantitative analysis of root system parameters including disease severity index (DSI), root length (cm/plant), fresh weight of root (mg/seedling), and fresh weight of lateral root (mg/seedling) comparing Xiaoheiqi (R) and L83-4752 (S). Data represent mean ± standard error. Asterisks indicate significant differences between resistant and susceptible accessions (* *p* < 0.05; ** *p* < 0.01), Student’s t-test. (**C**) Time-course analysis of antioxidant enzyme activities and osmoregulatory compound concentrations in root tissues following pathogen challenge. Upper panels show superoxide dismutase (SOD), peroxidase (POD), and catalase (CAT) activities (U mg^−1^ protein, μmol min^−1^ mg^−1^ protein, and μmol min^−1^ mg^−1^ protein, respectively). Lower panels display proline (Pro, μg g^−1^ fresh weight), soluble protein (mg g^−1^ fresh weight), and soluble sugar (mg g^−1^ fresh weight) concentrations. Treatment groups: S-CK (susceptible mock-inoculated, light gray), S-DT (susceptible *F. oxysporum*-inoculated, medium gray), R-CK (resistant mock-inoculated, light blue), R-DT (resistant *F. oxysporum*-inoculated, dark blue). Data represents standard means ± standard error from three biological replicates. Asterisks indicate significant differences between treatments (* *p* < 0.05, ** *p* < 0.01; ns = not significant) based on two-way ANOVA.

Initial screening of 381 soybean germplasm accessions identified two contrasting genotypes for *Fusarium oxysporum* resistance: the highly resistant landrace Xiaoheiqi and the susceptible line L83-4752. Following controlled inoculation with *F. oxysporum*, distinct phenotypic differences emerged between these accessions across the evaluation period (Figure 1A,B). The susceptible accession L83-4752 exhibited severe root and basal stem rot symptoms by 8 days post-inoculation (dpi), with progressive deterioration leading to a disease severity index (DSI) of 60.00 at 17 dpi. In contrast, Xiaoheiqi maintained healthy root architecture with minimal visible symptoms, resulting in a significantly lower DSI of 16.18 (*p* < 0.01). Root system analysis revealed that the resistant variety Xiaoheiqi exhibited significantly enhanced root development compared to the susceptible line L83-4752 across multiple parameters. Root length measurements showed that Xiaoheiqi produced substantially longer roots, reaching approximately 17 cm per plant compared to 11 cm per plant in L83-4752, representing a 55% increase (*p* < 0.05). Total root fresh weight was markedly higher in Xiaoheiqi at approximately 1.3 mg per seedling versus 0.6 mg per seedling in L83-4752, demonstrating a 117% increase in root biomass (*p* < 0.01). Lateral root development followed a similar pattern, with Xiaoheiqi producing approximately 0.8 mg per seedling of lateral root fresh weight compared to 0.4 mg per seedling in L83-4752, representing a 100% increase (*p* < 0.01). These results indicate that the resistant variety not only exhibits lower disease severity but also maintains superior root system architecture and biomass accumulation, suggesting a correlation between enhanced root development and disease resistance in soybean response to *Fusarium oxysporum* infection.

To investigate the differential responses between resistant and susceptible soybean varieties, we inoculated Xiaoheiqi (resistant) and L83-4752 (susceptible) plants with *F. oxysporum* and monitored disease progression and physiological parameters at 8, 11, 14, and 17 days post-inoculation (dpi). Four treatment groups were established: resistant mock control (R-CK), resistant *F. oxysporum* treatment (R-DT), susceptible mock control (S-CK), and susceptible *F. oxysporum* treatment (S-DT). Mock-inoculated controls (CK) received sterile water, while treatment groups (DT) were inoculated with *F. oxysporum* spore suspension. After inoculation with *F. oxysporum*, the activities of superoxide dismutase (SOD), peroxidase (POD), and catalase (CAT) in the roots of the resistant accession Xiaoheiqi significantly increased at all time points. Specifically, at 8 dpi post-inoculation, the SOD and POD activities in L83-4752 were significantly lower than those in Xiaoheiqi. At 17 dpi post-inoculation, the CAT activity in L83-4752 was also markedly lower compared to Xiaoheiqi. Furthermore, with the exception of proline content, which showed no significant difference between the two materials at 14 dpi post-inoculation, the other key osmoregulatory substances exhibited significant differences at all remaining critical time points.

### 2.2. Comprehensive Transcriptomic Profiling Reveals Defense-Associated Gene Expression Networks

To elucidate the molecular mechanisms underlying the observed phenotypic and physiological differences, RNA-seq analysis was conducted on root samples collected at 8, 11, 14, and 17 dpi from both inoculated and mock-inoculated plants. A total of 48 libraries were sequenced, generating 328.8 million high-quality paired-end reads with an average of 6.85 billion reads per library (Table S1). Quality assessment revealed high mapping rates to the soybean reference genome, with 96.52% of reads successfully aligned (range: 95.59–97.43%) and GC content between 42.89 and 43.99% (Table S2).

**Figure 2:**
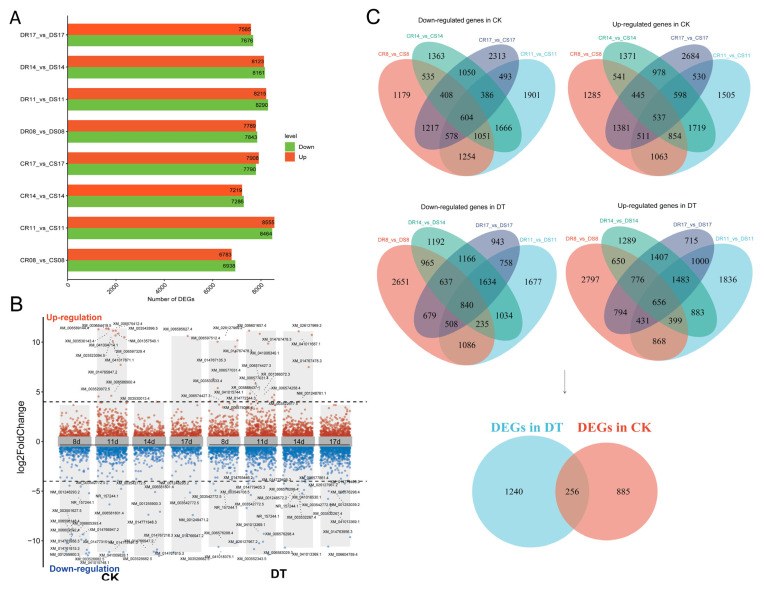
**Differential gene expression analysis and identification of key resistance-associated genes.** (**A**) Bar chart showing the number of differentially expressed genes (DEGs) identified in pairwise comparisons between resistant (R) and susceptible (S) genotypes at four time points (8d, 11d, 14d, 17d) under mock-inoculated (CK) and *F. oxysporum*-inoculated (DT) conditions. Green bars represent upregulated genes; red bars represent downregulated genes. Sample comparisons: CR vs. CS (resistant vs. susceptible mock-inoculated), DR vs. DS (resistant vs. susceptible pathogen-inoculated). (**B**) Volcano plots displaying highly differentially expressed genes (HDEGs) with stringent filtering criteria (|log_2_FC| ≥ 4, *p* < 0.01). Red dots indicate upregulated genes; blue dots indicate downregulated genes, with 67 key genes identified across the expression spectrum. Gene IDs are labeled for the most significantly altered transcripts. (**C**) Venn diagram analysis showing the relationships between DEG sets across time points and treatments. Upper panels show overlap patterns for downregulated (left) and upregulated (right) genes in mock-inoculated comparisons (CK). Lower panels display similar analysis for pathogen-inoculated comparisons (DT. The bottom panel shows the relationship between CK and DT gene sets.

For analytical clarity, samples were designated as follows: CR group (mock-inoculated Xiaoheiqi), CS group (mock-inoculated L83-4752), DR group (*F. oxysporum*-inoculated Xiaoheiqi), and DS group (*F. oxysporum*-inoculated L83-4752). Differential expression analysis identified substantial transcriptomic reprogramming following pathogen challenge (Figure 2A). In genotype comparisons without pathogen stress (CR vs. CS), 13,721 to 17,019 differentially expressed genes (DEGs) were detected across time points, reflecting inherent genetic differences between accessions. Following *F. oxysporum* inoculation (DR vs. DS), 15,261 to 16,505 DEGs were identified, indicating pathogen-responsive transcriptional changes.

To focus on the most significantly altered genes, stringent filtering criteria (|log2FC| ≥ 4, *p* < 0.01) identified 67 highly differentially expressed genes (HDEGs) distributed across the expression spectrum (Figure 2B). Venn diagram analysis revealed the relationships between gene sets across treatment conditions (Figure 2C). In mock-inoculated comparisons (CK group), 1141 DEGs were consistently identified across all four time points, comprising 537 upregulated and 604 downregulated genes. Following pathogen inoculation (DT group), 1496 DEGs showed consistent differential expression, including 656 upregulated and 840 downregulated genes. Notably, 256 DEGs were common to both treatment conditions, suggesting constitutive expression differences between genotypes, while 1240 and 885 DEGs were unique to pathogen-treated and mock-treated comparisons, respectively.

### 2.3. Expression Pattern Analysis Reveals Coordinated Transcriptional Responses

**Figure 3:**
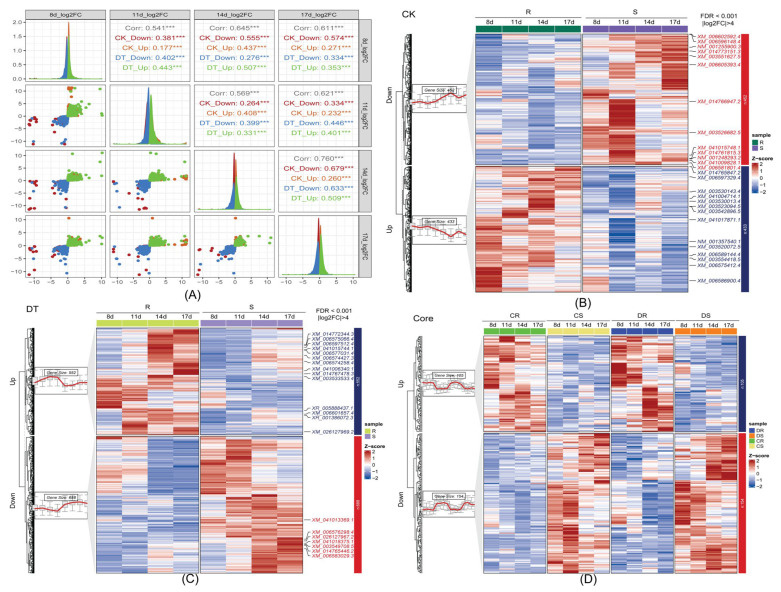
**Temporal expression dynamics and clustering analysis of differentially expressed genes between resistant and susceptible soybean genotypes.** (**A**) Correlation analysis and distribution plots showing the relationship between gene expression patterns in mock-inoculated (CK) and *F. oxysporum*-inoculated (DT) comparisons across four time points (8d, 11d, 14d, 17d). Scatter plots display log2 fold-change values with correlation coefficients and gene counts for upregulated and downregulated genes at each time point. Density plots show the distribution of expression changes, with consistent patterns indicating coordinated transcriptional responses. (**B**) Heatmap showing expression patterns of 1141 differentially expressed genes consistently identified across all time points in mock-inoculated comparisons (CK group), with hierarchical clustering revealing two main expression clusters corresponding to upregulated (537 genes) and downregulated (604 genes) gene sets. (**C**) Heatmap displaying expression patterns of 1496 differentially expressed genes consistently identified in pathogen-inoculated comparisons (DT group), clustered into upregulated (656 genes) and downregulated (840 genes) groups. (**D**) Core gene set analysis showing expression patterns of 256 genes that are differentially expressed in both CK and DT conditions, representing constitutive differences between resistant and susceptible genotypes that are maintained regardless of pathogen presence. Color scale indicates log2 fold-change values (blue = downregulated, red = upregulated). Sample labels: R = resistant (Xiaoheiqi), S = susceptible (L77-2061), with time points at 8d, 11d, 14d, and 17d post-treatment. Asterisks denote a statistically significant differences indicated by Student’s t-test (***, *p* < 0.001).

To characterize temporal expression dynamics, K-means clustering analysis was performed on DEG sets from both treatment groups (Figure 3A–C). Expression pattern comparison using log2-fold change values demonstrated consistent spatial distribution across time points, with downregulated DEGs clustering in the third quadrant and upregulated DEGs in the first quadrant, indicating coordinated transcriptional responses before and after pathogen challenge. Independent K-means clustering generated two optimal clusters for each treatment group. In the CK group, 885 DEGs were divided into clusters containing 452 downregulated and 433 upregulated genes. The DT group’s 1240 DEGs formed clusters of 688 downregulated and 552 upregulated genes (Figure 3B). The 256 common DEGs between treatment groups exhibited consistent expression patterns (Figure 3C), suggesting these genes represent fundamental expression differences between resistant and susceptible genotypes that are maintained regardless of pathogen presence.

### 2.4. Functional Enrichment Analysis Identifies Key Defense Pathways

**Figure 4:**
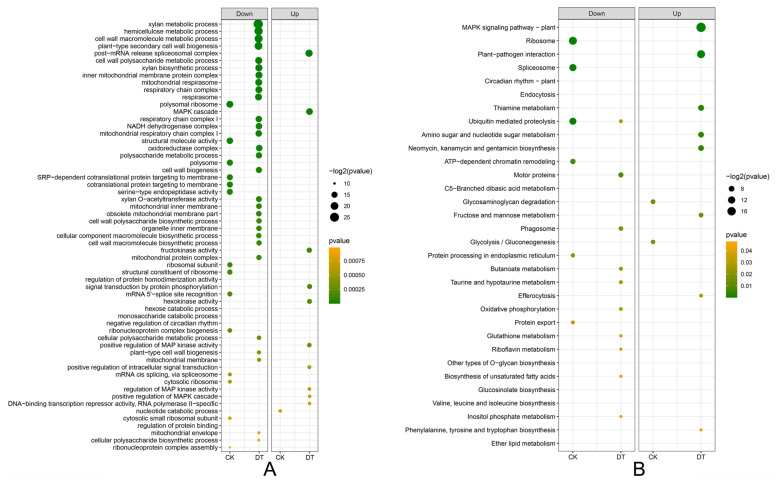
Functional enrichment of DEGs. (**A**): GO enrichment analysis of the DEGs (up- and down-regulation) in R relative to S based on the GO database by filter *p* value < 0.001. The negative log *p* value was assigned to x-axis and GO term was assigned to the y-axis. Bubble area was positively proportional to the significant of genes in a certain GO term; (**B**): KEGG enrichment analysis of the DEGs (up- and down-regulation) in R relative to S based on the KEGG database filter *p* value < 0.05. The negative log *p* value was assigned to x-axis and pathway was assigned to the y-axis. Bubble area was positively proportional to the significant genes in a certain pathway.

Gene Ontology (GO) and Kyoto Encyclopedia of Genes and Genomes (KEGG) pathway enrichment analyses were conducted to identify biological processes and metabolic pathways associated with disease resistance (Figure 4A,B). GO functional annotation of the 885 DEGs in genotype comparisons without pathogen (CR vs. CS) revealed limited enrichment, with downregulated DEGs enriched in 14 terms and upregulated DEGs in only one term (GO:0009166, nucleotide catabolic process). In contrast, pathogen-challenged comparisons (DR vs. DS) showed substantial functional enrichment, with downregulated DEGs enriched in 29 terms and upregulated DEGs in 10 terms, indicating extensive transcriptional reprogramming during host–pathogen interactions. KEGG pathway analysis provided deeper insights into resistance mechanisms (Figure 4B). In mock-inoculated comparisons, downregulated DEGs enriched 6 pathways while upregulated DEGs enriched 8 pathways. Notably, pathogen-challenged samples showed significant enrichment in defense-related pathways, particularly the MAPK signaling pathway–plant (ko04016) and plant–pathogen interaction (ko04626), suggesting their central roles in resistance responses. Detailed examination of these critical pathways revealed eight DEGs co-enriched in both MAPK signaling and plant–pathogen interaction pathways: *Glyma.04G253500*, *Glyma.05G123200*, *Glyma.05G198700*, *Glyma.07G066800*, *Glyma.10G178400*, *Glyma.13G084100*, *Glyma.16G214500*, and *Glyma.18G236800* (Table S3). Functional annotation indicated that the encoding of these genes corresponds to their roles in defense signal cascades (Table S4).

### 2.5. Validation and Identification of GmCML as a Key Resistance Gene

**Figure 5:**
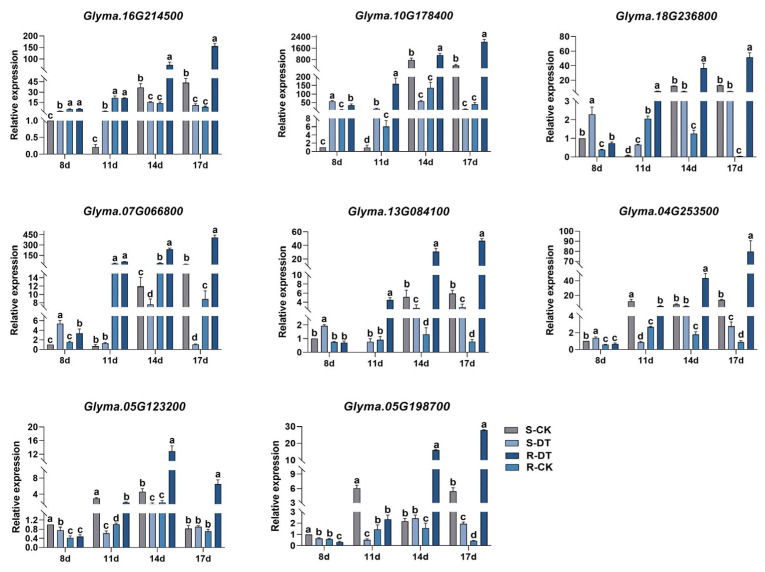
**Quantitative RT-PCR validation of eight key differentially expressed genes identified from RNA-seq analysis.** Expression profiles of candidate genes (*Glyma.16G214500*, *Glyma.10G178400*, *Glyma.18G236800*, *Glyma.07G066800*, *Glyma.13G084100*, *Glyma.04G253500*, *Glyma.05G123200*, *and Glyma.05G198700*) that were co-enriched in both MAPK signaling pathway-plant and plant–pathogen interaction pathways. Gene expression was analyzed at 8, 11, 14, and 17 days post-inoculation across four treatment groups: S-CK (susceptible mock-inoculated, light gray), S-DT (susceptible *F. oxysporum*-inoculated, medium gray), R-DT (resistant *F. oxysporum*-inoculated, dark blue), and R-CK (resistant mock-inoculated, light blue). Relative expression levels were calculated using the 2^(−ΔΔCt) method with *GmActin* as the reference gene. Data represent means ± standard error from three biological replicates with three technical replicates each. Different letters indicate significant differences among treatments at each time point (*p* < 0.05, one-way ANOVA followed by Tukey’s HSD test).

To validate RNA-seq findings and identify the most promising candidate genes, quantitative real-time PCR (qRT-PCR) analysis was performed on the eight co-enriched DEGs (Figure 5). Expression patterns determined by qRT-PCR showed strong correlation with RNA-seq data across all tested genes, confirming the reliability of transcriptomic analysis. Among these candidates, *Glyma.10G178400* exhibited the most dramatic expression differences between resistant and susceptible genotypes following pathogen challenge. Specifically, *Glyma.10G178400* showed progressive upregulation in the resistant genotype (DR group) across the time course, reaching peak expression at 17 dpi. At this time point, relative expression levels in resistant plants were approximately 185-fold higher than in susceptible plants, representing the largest expression differential among all tested genes. The gene showed minimal expression in susceptible plants throughout the evaluation period, while maintaining consistent upregulation in resistant plants from 11 dpi onward. Sequence analysis revealed that *Glyma.10G178400* encodes a 151-amino acid protein containing characteristic EF-hand calcium-binding domains, classifying it as a calmodulin-like (CML) protein. Based on its dramatic differential expression and functional annotation, this gene was designated *GmCML* and selected for comprehensive functional characterization.

### 2.6. Functional Validation Demonstrates GmCML’s Role in Disease Resistance

**Figure 6:**
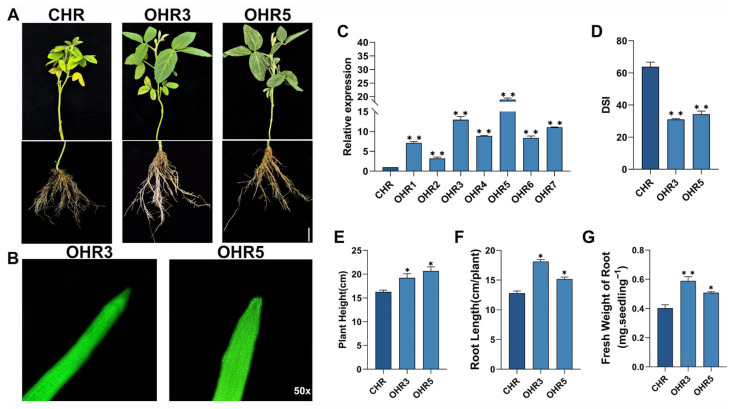
***GmCML* overexpression improves soybean resistance to *Fusarium oxysporum* through transgenic hairy root analysis.** (**A**) Typical images of control hairy roots (CHRs) and two GmCML-overexpressing lines (OHR3, OHR5) at 17 days after inoculation with *F. oxysporum*, showing both shoot and root systems. Scale bar = 2 cm. (**B**) Fluorescence microscopy confirms transgenic hairy roots expressing GFP marker gene in OHR3 and OHR5 lines under UV light (50× magnification). (**C**) qRT-PCR validates *GmCML* expression levels in seven independent overexpression lines (OHR1–OHR7) compared to control hairy roots (CHRs). Data show fold-change relative to CHR with *GmActin* as reference gene. (**D**) Disease severity index (DSI) assessment at 17 days after inoculation, showing significantly reduced disease symptoms in *GmCML*-overexpressing lines. (**E**) Plant height measurements reveal improved growth maintenance in overexpressing lines following pathogen challenge. (**F**) Root length measurement shows better root system development in transgenic lines. (**G**) Fresh weight of root systems indicates increased biomass in *GmCML*-overexpressing hairy roots. All physiological data are presented as means ± standard error from at least five independent hairy root samples per treatment. Asterisks denote significant differences compared to CHR control (* *p* < 0.05, ** *p* < 0.01) according to Student’s t-test.

**Figure 7:**
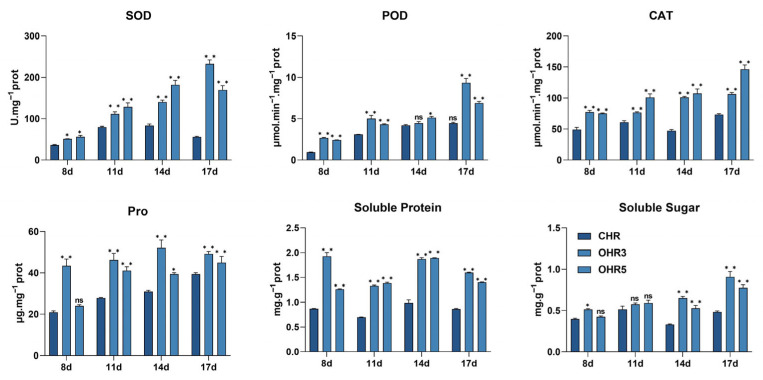
**Enhanced antioxidant enzyme activities and osmoregulatory compound accumulation in *GmCML*-overexpressing hairy roots following pathogen challenge.** Comparative analysis of physiological parameters in control hairy roots (CHRs, dark blue) and two *GmCML*-overexpressing lines (OHR3 and OHR5, medium and light blue) at 8, 11, 14, and 17 days post-inoculation with *F. oxysporum*. Upper panels show antioxidant enzyme activities: superoxide dismutase (SOD, U mg^−1^ protein), peroxidase (POD, μmol min^−1^ mg^−1^ protein), and catalase (CAT, μmol min^−1^ mg^−1^ protein). Lower panels display osmoregulatory compound concentrations: proline (Pro, μg g^−1^ fresh weight), soluble protein (mg g^−1^ fresh weight), and soluble sugar (mg g^−1^ fresh weight). Data represent means ± standard error from three biological replicates with three technical replicates each. Asterisks indicate significant differences between overexpression lines and control (* *p* < 0.05, ** *p* < 0.01) based on one-way ANOVA followed by Tukey’s HSD test. ns = not significant.

To investigate *GmCML’s* biological function in disease resistance, transgenic soybean hairy roots overexpressing this gene were generated using *Agrobacterium rhizogenes*-mediated transformation. Seven independent overexpression lines (OHR1-OHR7) were obtained, with *GmCML* transcript levels ranging from 5.1-fold to 20.6-fold higher than control hairy roots (CHRs) as determined by qRT-PCR analysis. Two high-expression lines, OHR3 and OHR5, were selected for detailed phenotypic and physiological analysis. Following controlled inoculation with *F. oxysporum*, *GmCML*-overexpressing hairy roots exhibited significantly enhanced disease resistance compared to control lines (Figure 6A–C). Disease severity assessments revealed that OHR3 and OHR5 lines maintained lower disease indices (31.02 and 34.23, respectively) compared to control hairy roots (63.89) at 17 dpi. Morphological measures demonstrated that overexpression lines retained greater root length (OHR3: 18.13 cm, OHR5: 15.15 cm) compared to controls (12.76 cm), while root fresh weight was similarly enhanced in overexpressing lines (OHR3: 0.59 g, OHR5: 0.50 g) versus controls (0.40 g). Phenotypic resistance of *GmCML* hairy roots to *Fusarium oxysporum* prompted further physiological and biochemical analyses. Evaluation of antioxidant enzyme activities and osmolyte contents revealed that all lines exhibited significant increases in SOD, POD, and CAT activities, as well as in proline, soluble sugar, and soluble protein levels after inoculation with *Fusarium oxysporum*. These increases were more pronounced in the *GmCML*-OHR line. These results suggest that *GmCML* enhances resistance to pathogen infection by boosting intracellular antioxidant enzyme activities and accumulating compatible solutes, thereby reducing root damage (Figure 7).

### 2.7. Population Genetic Analysis Reveals Natural Variation in GmCML

**Figure 8:**
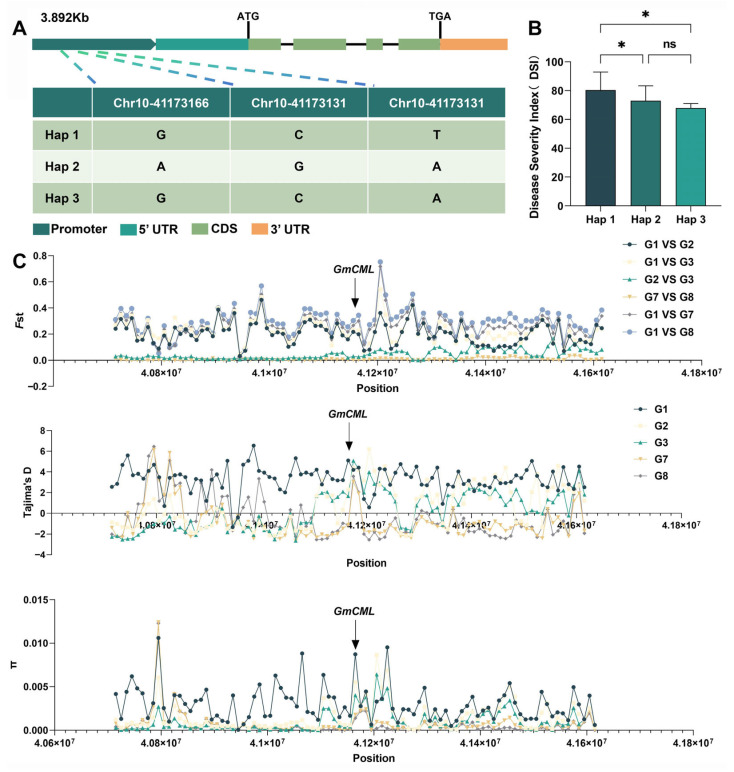
**Haplotype diversity and evolutionary signatures of *GmCML* in soybean populations**. (**A**) Schematic representation of the *GmCML* gene structure showing the positions of three key SNPs in the promoter region that define haplotype groups. (**B**) Disease resistance comparison among *GmCML* haplotypes in 295 soybean accessions. (**C**) Population genomics analysis showing selection signatures across the *GmCML* genomic region. Population codes: G1 = wild soybean, G2 = Huang-Huai-Hai landraces, G3 = Huang-Huai-Hai cultivars, G7 = Northeast China landraces, G8 = Northeast China cultivars. Asterisks above bars denote a statistically significant differences indicated by Student’s t-test (*, *p* < 0.05, ns, *p* > 0.05).

To investigate the relationship between natural *GmCML* variation and disease resistance phenotypes, haplotype analysis was conducted using genomic resequencing data from 295 diverse soybean accessions. Sequence analysis identified 26 single-nucleotide polymorphisms (SNPs) within the *GmCML* gene region, with three SNPs located in the promoter region showing significant association with resistance phenotypes. Based on these three promoter SNPs, the germplasm collection was classified into three distinct haplotypes (Figure 8A). Haplotype 1 (Hap1) represented the most common variant (*n* = 142, 48.1%), while Haplotype 2 (Hap2, *n* = 89, 30.2%) and Haplotype 3 (Hap3, *n* = 64, 21.7%) showed lower frequencies. Phenotypic evaluation revealed significant differences in disease resistance among haplotypes (Figure 8B). Both Hap2 and Hap3 exhibited significantly lower disease indices compared to Hap1. Although Hap3 showed numerically lower disease severity than Hap2, this difference was not statistically significant.

### 2.8. Evolutionary Analysis Indicates Selection During Domestication

To investigate whether *GmCML* has been subject to selection pressure during soybean domestication, population genetic analysis was conducted using publicly available genomic data from the SoyMD database. This analysis included 591 wild soybean accessions, 439 landraces from the Huang-Huai-Hai region, 379 cultivars from the Huang-Huai-Hai region, 539 landraces from Northeast China, and 842 cultivars from Northeast China. Fixation index (*F*st) analysis revealed moderate but significant differentiation between wild and domesticated populations in the *GmCML* genomic region (*F*st = 0.20–0.30), indicating selective pressure during domestication. Nucleotide diversity (π) values showed reduced variation in cultivated varieties compared to wild populations, consistent with selection for favorable alleles. Tajima’s D values in the *GmCML* region were significantly negative in cultivated of northern China (G8, −0.45), providing additional evidence for positive selection. Although the selection signal was relatively modest compared to other major domestication genes, these results suggest that *GmCML*-mediated disease resistance has contributed to adaptive evolution during soybean domestication and improvement. The maintenance of multiple haplotypes in modern cultivars indicates that genetic diversity at this locus continues to provide adaptive value for disease resistance in diverse environmental conditions.

## 3. Discussion

This study provides comprehensive insights into the molecular mechanisms underlying soybean resistance to *Fusarium oxysporum* root rot through the identification and functional characterization of *GmCML*, a calmodulin-like protein that serves as a critical regulator of plant immunity. The integration of transcriptomic profiling, functional validation, and population genetics approaches reveals novel aspects of calcium-mediated defense signaling while addressing fundamental questions about crop disease resistance mechanisms.

### 3.1. GmCML Functions as a Master Regulator of Calcium-Mediated Defense Responses

The dramatic upregulation of *GmCML* in resistant genotypes positions this gene as a key molecular switch in soybean–*Fusarium* interactions. This expression pattern aligns with established models of plant immunity, where rapid and sustained gene expression changes are hallmarks of effective defense responses [43,44]. The calmodulin-like protein structure of *GmCML*, containing characteristic EF-hand calcium-binding domains, suggests it functions as a calcium sensor that translates pathogen-induced Ca^2+^ fluctuations into downstream signaling events. The functional validation through hairy root overexpression provides strong evidence for *GmCML*’s role in disease resistance. The coordinated activation of multiple antioxidant enzymes (SOD, POD, CAT) and the accumulation of osmoregulatory compounds demonstrate that *GmCML* orchestrates a complex, multi-layered defense strategy. This comprehensive response likely offers both immediate protection against pathogen-induced oxidative stress and longer-term cellular resilience during sustained infection pressure [45,46]. However, the mechanism by which *GmCML* specifically regulates these downstream processes remains unclear. Unlike well-characterized calmodulin proteins that directly interact with target enzymes, the specific protein–protein interactions and transcriptional targets of *GmCML* need further investigation. The observation that antioxidant enzyme activities increase significantly in overexpressing lines suggests either direct enzymatic regulation or transcriptional activation of enzyme-encoding genes.

### 3.2. Integration with Plant Defense Signaling Networks

The enrichment of differentially expressed genes in MAPK signaling and plant–pathogen interaction pathways supports current models of plant immunity that emphasize interconnected signaling networks rather than linear pathways [43,47]. The identification of eight genes co-enriched in both pathways, including *GmCML*, suggests these represent key nodes where multiple defense signals converge. The temporal expression dynamics of *GmCML*, with peak expression occurring at 17 dpi, indicate that this gene functions during sustained defense responses rather than initial pathogen recognition. This timing suggests *GmCML* may be particularly important for maintaining resistance during prolonged infection periods when initial defense responses begin to wane. Such sustained expression patterns are characteristic of genes involved in induced systemic resistance and immune memory [48,49]. The moderate selection signal detected during soybean domestication provides evolutionary context for *GmCML* function. While not as strong as signals associated with major domestication traits, the positive selection evidence suggests that *GmCML*-mediated resistance has provided adaptive advantages during crop improvement. The maintenance of multiple functional haplotypes in modern cultivars indicates that genetic diversity at this locus continues to provide value under diverse environmental conditions.

### 3.3. Physiological Integration of Defense Responses

The coordinated activation of antioxidant systems and osmoregulatory processes by *GmCML* reflects a sophisticated integration of cellular defense mechanisms. Reactive oxygen species (ROS) accumulation typically occurs during pathogen infection, acting both as antimicrobial agents and signaling molecules [50,51]. The increased activities of SOD, POD, and CAT in *GmCML*-overexpressing lines suggest that this protein helps maintain optimal ROS balance during infection. The concurrent buildup of proline, soluble sugars, and proteins indicates that *GmCML* also regulates osmotic adjustment mechanisms. These compounds have multiple roles, including stabilizing proteins, protecting membranes, and regulating metabolism during stress [52,53]. The integration of antioxidant and osmoregulatory responses via a single regulatory protein provides an efficient way to coordinate complex cellular adaptations. However, the specific biochemical mechanisms are still unclear. *GmCML* could influence enzyme activities through protein interactions, and it acts as a transcriptional regulator [54,55]. The notable increases in enzyme activities imply transcriptional regulation might be involved, but this needs to be confirmed through gene expression analysis of relevant enzyme-encoding genes.

### 3.4. Broader Implications for Plant Immunity Research

This study contributes to growing evidence that calcium signaling plays central roles in plant immunity beyond the well-characterized early defense responses. The identification of *GmCML* as a regulator of sustained defense responses expands our understanding of how plants maintain resistance during prolonged pathogen challenges. This has broader implications for understanding durable resistance mechanisms that remain effective over multiple growing seasons. The integration of transcriptomic and functional approaches demonstrates the value of comprehensive gene characterization studies. While high-throughput omics technologies can identify candidate genes, functional validation remains essential for understanding biological mechanisms and assessing practical applications. The population genetic analysis adds evolutionary context that helps interpret the biological significance of identified variants.

## 4. Materials and Methods

### 4.1. Plant Materials and Growth Conditions

Soybean (*Glycine max* (L.) Merr.) germplasm accessions used in this study were obtained from the National Mid-term Genebank for Cold region Crops and Soybean, Heilongjiang Academy of Agricultural Sciences, Harbin, China. Two contrasting genotypes were selected based on previous large-scale screening of 381 germplasm accessions: Xiaoheiqi and L83-4752 [30]. For transformation experiments, soybean cultivar DN50 was used due to its high transformation efficiency. Seeds were surface sterilized with 75% ethanol for 30 s, followed by 2% sodium hypochlorite solution for 10 min, and rinsed five times with sterile distilled water. Sterilized seeds were germinated on moist filter paper in Petri dishes at 25 °C in darkness for 48 h before transplanting.

### 4.2. Pathogen Culture and Inoculum Preparation

*Fusarium oxysporum*, which was originally isolated from infected soybean roots in Heilongjiang Province, was provided by the Institute of Plant Protection at Heilongjiang Academy of Agricultural Sciences. Pathogen identity was confirmed through morphological characterization and ITS sequence analysis. The pathogen was initially cultured on potato dextrose agar (PDA) medium and incubated at 26 °C in the dark for 7 days to activate it. Inoculum was prepared using a modified sorghum grain method. Autoclaved sorghum grains (500 g) were placed in 1 L flasks and inoculated with 5 mL of conidial suspension (1 × 10^6^ conidia/mL). The inoculated grains were incubated at 26 °C with daily mixing for 17 days until complete colonization. Conidial concentration was determined using a hemocytometer, and viability was confirmed by plating serial dilutions on PDA. Only batches with >95% germination rates were used for plant inoculation experiments.

### 4.3. Disease Resistance Evaluation

Disease resistance assessment was conducted following established protocols by Trivedi et al. [56], with modifications. Sterilized vermiculite was filled into plastic pots (11 cm height, 8 cm diameter) to two-thirds capacity. Seeds were surface-sterilized and treated with carbendazim fungicide (50% WP, Jiangsu Kwin Group Co., Ltd., Nanjing, China) at a concentration of 0.2% (*w*/*v*) for 30 min prior to planting. The fungicide was thoroughly mixed into the vermiculite at a concentration of 4% (*w*/*w*) to ensure uniform pathogen distribution. An additional 0.5 cm layer of sterile vermiculite was applied as top dressing. For each treatment, 15 surface-sterilized soybean seeds were sown per pot at 1 cm depth and wholly covered with dry sterile vermiculite. Mock-inoculated controls used autoclaved sorghum grains without a pathogen. Each treatment was replicated three times, and experiments were conducted twice independently. Plants were grown in a controlled-environment growth chamber (Conviron PGR15, Manitoba, Canada) maintained at 25 ± 2 °C with a 14 h photoperiod (200 μmol m^−2^ s^−1^ photosynthetic photon flux density) provided by LED lights. Relative humidity was maintained at 65 ± 5%, and plants were watered every two days with sterile distilled water. Disease severity was evaluated at 14 days post-inoculation using a modified 0–7 rating scale: 0 = no symptoms, normal root growth; 1 = slight browning of primary root, normal lateral roots; 3 = pronounced blackening of primary root with reduced lateral roots; 5 = severe primary root blackening with significantly reduced lateral roots and impaired shoot growth; 7 = plant death or failure to emerge. The disease severity index (DSI) was calculated as: DSI = [Σ(disease rating × number of plants in each category)/(total number of plants × highest disease rating)] × 100.

### 4.4. Physiological and Biochemical Parameter Analysis

Root samples were collected at 8, 11, 14, and 17 days post-inoculation from both inoculated and mock-inoculated plants. For each time point, three biological replicates were collected, with each replicate consisting of pooled root tissues from five plants. Samples were immediately frozen in liquid nitrogen and stored at −80 °C until analysis.

#### 4.4.1. Antioxidant Enzyme Activity Assays

Superoxide dismutase (SOD) activity was determined using a commercial assay kit (AKAO001M-100S, Boxbio, Beijing, China) based on nitro blue tetrazolium (NBT) reduction inhibition. Root tissues (100 mg) were homogenized in 1 mL of ice-cold extraction buffer (50 mM phosphate buffer, pH 7.8, containing 1 mM EDTA and 1% polyvinylpolypyrrolidone). The homogenate was centrifuged at 12,000× *g* for 20 min at 4 °C. The reaction mixture contained 20 μL enzyme extract, 20 μL NBT solution, 20 μL riboflavin solution, and 140 μL assay buffer in a 96-well plate. After illumination under fluorescent light (4000 lux) for 30 min at 25 °C, absorbance was measured at 560 nm using a microplate reader (BioTek Synergy H1, Winooski, VT, USA). Peroxidase (POD) activity was measured using a detection kit (AKAO005M, Boxbio, Beijing, China) based on guaiacol oxidation. Root tissue (100 mg) was ground in 1 mL of extraction buffer (50 mM phosphate buffer, pH 7.0) and centrifuged at 12,000× *g* for 15 min at 4 °C. The reaction mixture contained 20 μL enzyme extract, 20 μL guaiacol substrate, 20 μL hydrogen peroxide, and 140 μL reaction buffer. Absorbance was recorded at 470 nm at 30 s (A_1_) and 90 s (A_2_), with POD activity calculated using ΔA = A_2_ − A_1_. Catalase (CAT) activity was determined using a corresponding kit (AKAO003-2M, Boxbio, Beijing, China) by monitoring hydrogen peroxide decomposition. Root tissue (100 mg) was homogenized in 1 mL extraction buffer (50 mM phosphate buffer, pH 7.0, containing 1 mM EDTA) and centrifuged at 12,000× *g* for 15 min at 4 °C. The reaction mixture contained 20 μL enzyme extract and 180 μL substrate solution (20 mM H_2_O_2_) in UV-transparent 96-well plates. Absorbance at 240 nm was recorded at 5 and 65 s after mixing.

#### 4.4.2. Osmoregulatory Compound Analysis

Proline content was quantified using a commercial assay kit (AKAM003M, Boxbio, Beijing, China) based on the ninhydrin reaction. Root tissue (100 mg) was homogenized in 1 mL of 3% aqueous sulfosalicylic acid and heated in boiling water for 10 min. After cooling and centrifugation at 10,000× *g* for 10 min, the reaction mixture contained 100 μL supernatant, 100 μL glacial acetic acid, 100 μL ninhydrin reagent, and 200 μL toluene. Absorbance was measured at 520 nm after 10 min of incubation. Soluble sugar content was evaluated using a plant soluble sugar assay kit (AKPL008M, Boxbio, Beijing, China) employing the anthrone method. Root tissue (100 mg) was ground in 1 mL of distilled water and heated at 95 °C for 10 min in sealed tubes. After cooling and centrifugation at 8000× *g* for 10 min, 20 μL supernatant was mixed with 200 μL anthrone reagent (0.2% anthrone in concentrated sulfuric acid). The mixture was heated at 95 °C for 10 min, and the absorbance was measured at 620 nm. Soluble protein content was determined using a Bradford protein assay kit (SP29721, Spbio, Wuhan, China). Root tissue (100 mg) was homogenized in 1 mL of 0.9% NaCl solution and centrifuged at 10,000× *g* for 10 min at 4 °C. Following standard ELISA procedures with biotin-labeled antibody and streptavidin–HRP conjugate, absorbance was measured at 450 nm.

### 4.5. RNA Extraction and Quality Assessment

Total RNA was extracted from root tissues using TRIzol reagent (Invitrogen, Carlsbad, CA, USA) following the manufacturer’s protocol. Root samples (100 mg) were ground to fine powder in liquid nitrogen using a mortar and pestle. The powder was immediately mixed with 1 mL TRIzol reagent and incubated at room temperature for 5 min. Following chloroform extraction and isopropanol precipitation, RNA pellets were washed twice with 75% ethanol and dissolved in RNase-free water. RNA quality and integrity were assessed using multiple methods. RNA concentration was measured using a NanoDrop 2000 spectrophotometer (Thermo Fisher Scientific, Waltham, MA, USA), with purity assessed by A260/A280 and A260/A230 ratios. RNA integrity was evaluated using an Agilent 2100 Bioanalyzer (Agilent Technologies, Santa Clara, CA, USA), with RNA integrity numbers (RIN) calculated. Only RNA samples with RIN values > 7.0, A260/A280 ratios between 1.8 and 2.2, and A260/A230 ratios > 1.8 were used for downstream applications.

### 4.6. RNA Sequencing and Data Processing

Transcriptomic analysis was performed with reference to the methodology described by Hohenfeld et al. [57]. cDNA libraries were constructed using the NEBNext Ultra RNA Library Prep Kit for Illumina (NEB, Beijing, China) according to the manufacturer’s instructions. mRNA was purified from total RNA using oligo(dT) magnetic beads, followed by fragmentation using divalent cations under elevated temperature. First-strand cDNA synthesis was performed using random hexamer primers and reverse transcriptase, followed by second-strand cDNA synthesis using DNA polymerase I and RNase H. cDNA fragments were end-repaired, A-tailed, and ligated with indexed adapters. Library quality was assessed using an Agilent 2100 Bioanalyzer, and concentration was determined by qPCR using a KAPA Library Quantification Kit (Kapa Biosystems, Wilmington, MA, USA). Libraries were pooled in equimolar ratios and sequenced on an Illumina HiSeq X Ten platform, generating 150 bp paired-end reads. Raw sequencing reads were processed using FastQC v0.11.9 for quality assessment. Low-quality reads and adapter sequences were removed using Trimmomatic v0.39 with parameters: LEADING:20 TRAILING:20 SLIDINGWINDOW:4:20 MINLEN:50. Clean reads were mapped to the soybean reference genome (*Glycine max* Wm82.a4.v1) using STAR aligner v2.7.10a with default parameters. Gene expression levels were quantified using RSEM v1.3.2, calculating Fragments Per Kilobase of exon per Million fragments mapped (FPKM) values.

### 4.7. Differential Expression Analysis

Differential gene expression analysis was performed using the edgeR package v3.36.0 in R v4.1.0. Raw count matrices were filtered to remove lowly expressed genes (genes with <1 CPM in at least three samples), and library sizes were normalized using the trimmed mean of M-values (TMM) method. Biological coefficient of variation was estimated using empirical Bayes methods, and differential expression was tested using exact tests based on the negative binomial distribution. Statistical significance was determined using false discovery rate (FDR) correction with the Benjamini–Hochberg method [58]. Genes with |log_2_FC| ≥ 1, FDR < 0.05, and *p* < 0.05 were considered significantly differentially expressed. For highly differentially expressed genes (HDEGs), more stringent criteria were applied: |log_2_FC| ≥ 4 and *p* < 0.01.

### 4.8. Functional Enrichment Analysis

Gene Ontology (GO) functional annotation was performed using the cluster Profiler package v4.2.2 in R. Differentially expressed gene lists were mapped to GO terms using the org.Gmax.eg.db annotation package, and enrichment analyses were conducted for biological process, cellular component, and molecular function categories. Hypergeometric tests were performed to identify significantly overrepresented GO terms, with Benjamini–Hochberg correction applied (adjusted *p* < 0.05). KEGG pathway enrichment analysis was performed using the clusterProfiler package with the KEGG.db annotation database. Differentially expressed genes were mapped to KEGG pathways, and statistical significance assessed using hypergeometric tests with Benjamini–Hochberg correction. Pathways with adjusted *p* values < 0.05 were considered significantly enriched.

### 4.9. Quantitative Real-Time PCR Validation

Gene-specific primers for qRT-PCR were designed using Primer Premier 5.0 software with criteria: primer length 18–25 nucleotides, melting temperature 55–65 °C, GC content 40–60%, and amplicon length 80–200 bp (Table S5). Primer specificity was verified by BLAST(2.15.0+) analysis against the soybean genome. The soybean *Actin* gene (*GmActin*, *Glyma.18G290800*) was selected as the internal reference gene based on stable expression across treatments [3]. First-strand cDNA synthesis was performed using PrimeScript RT Master Mix (TaKaRa, Dalian, China). Total RNA (1 μg) was used as template in a 20 μL reaction containing 4 μL 5× PrimeScript RT Master Mix. The reaction was incubated at 37 °C for 15 min followed by 85 °C for 5 s. Synthesized cDNA was diluted to 500 ng/μL and stored at −20 °C. qRT-PCR reactions were performed using a Roche LightCycler 96 System with SYBR Premix Ex Taq II (TaKaRa, Shiga, Japan). Each 20 μL reaction contained 10 μL SYBR Premix, 0.5 μL each primer (10 μM), 2 μL cDNA template, and 7 μL nuclease-free water. Thermal cycling conditions were: 95 °C for 30 s, followed by 40 cycles of 95 °C for 15 s and 60 °C for 30 s. Melting curve analysis was performed from 60 °C to 95 °C with 0.5 °C increments. Relative gene expression was calculated using the 2^(−ΔΔCt) method with three biological and technical replicates.

### 4.10. GmCML Functional Characterization

PCR amplification was performed using KOD-Plus-Neo DNA polymerase (Toyobo, Osaka, Japan) with conditions: 94 °C for 2 min; 30 cycles of 98 °C for 10 s, 58 °C for 30 s, 68 °C for 90 s; final extension at 68 °C for 10 min. The amplified *GmCML* CDS was cloned into pCAMBIA3301 binary vector under CaMV 35S promoter control using standard molecular cloning techniques. The construct was verified by restriction enzyme digestion and Sanger sequencing. The overexpression vector pCAMBIA3301-*GmCML* was transformed into *Agrobacterium rhizogenes* strain K599 using the freeze–thaw method.

### 4.11. Hairy Root Transformation

Soybean hairy root transformation was performed using established protocols. *A. rhizogenes* K599 harboring pCAMBIA3301-*GmCML* was cultured in YEP medium supplemented with kanamycin (50 mg/L) at 28 °C with shaking (200 rpm) until OD_600_ reached 0.6–0.8. Bacterial cells were resuspended in sterile water to OD_600_ = 0.5. Seven-day-old DN50 seedlings with fully expanded cotyledons were used for transformation. Hypocotyl segments were wounded using a sterile scalpel and immersed in bacterial suspension for 10 min. After infection, seedlings were placed on sterile moist filter paper and incubated at 25 °C in darkness for 48 h. Hairy roots emerged 10–14 days post-infection. Transgenic roots were identified using stereo fluorescence microscopy to detect GFP expression and qRT-PCR analysis of *GmCML* expression levels.

### 4.12. Disease Resistance Assay in Hairy Roots

Transgenic hairy root systems with 2 transgenic roots(OHR3 and OHR5) were selected for disease resistance evaluation. Root systems were carefully removed from vermiculite, washed with sterile water, and transplanted into sterile vermiculite containing *F. oxysporum* inoculum. Control hairy roots transformed with wild type were included for comparison. Disease development was monitored daily, with final evaluation conducted at 17 days post-inoculation. Parameters measured included disease severity index, total root length, and fresh root weight. Root length was measured using ImageJ software (1.54g) from digital photographs, and fresh weight determined immediately after harvest. Physiological analyses were performed following the same protocols described above.

### 4.13. Population Genetic Analysis

#### 4.13.1. Haplotype Analysis

Haplotype analysis of the *GmCML* gene was performed across 295 Chinese soybean germplasm accessions. The *GmCML* gene sequence (including 2 kb upstream and 1 kb downstream regions) was extracted from the reference genome and used for BLAST analysis against the resequencing dataset. Single-nucleotide polymorphisms (SNPs) and insertion–deletion polymorphisms were identified using SAMtools/BCFtools v1.12 with quality filters: mapping quality > 20, base quality > 20, and minimum coverage > 5×. Haplotype reconstruction was performed using DnaSP v6.12.03 software, including only polymorphic sites with minor allele frequency > 5% [59].

#### 4.13.2. Analysis of Selective Sweep in the GmCML Gene During Soybean Domestication

Selection analysis was conducted using publicly available genomic data from the SoyMD database, including 591 wild soybean accessions, 439 landraces from the Huang-Huai-Hai region, 379 cultivars from the Huang-Huai-Hai region, 539 landraces from Northeast China, and 842 cultivars from Northeast China [60]. Population differentiation was assessed using fixation index (*F*st) calculations implemented in VCFtools v0.1.16. Nucleotide diversity (π) was calculated for sliding windows (window size 10 kb, step size 1 kb) using DnaSP v6.12.03. Tajima’s D values were calculated to detect deviations from neutral evolution. Selective sweep detection was performed using the composite likelihood ratio test implemented in SweeD v4.0.0.

### 4.14. Statistical Analysis

All experiments were conducted using completely randomized designs with appropriate replication. For transcriptomic analysis, three biological replicates were included for each treatment combination. Statistical analyses were performed using R v4.1.0 with appropriate packages. Data normality was assessed using Shapiro–Wilk tests, and homogeneity of variance was evaluated using Levene’s test. For normally distributed data, ANOVA was performed followed by Tukey’s HSD test for multiple comparisons. Non-parametric data were analyzed using Kruskal–Wallis tests followed by Dunn’s test with Bonferroni correction. Significance levels were set at *p* < 0.05 unless otherwise specified. Data are presented as means ± standard error of the mean.

## 5. Conclusions

Our findings revealed that following inoculation with *Fusarium oxysporum*, the difference in disease index between the highly resistant material Xiaoheiqi and the highly susceptible material L83-4752 was attributed to variations in antioxidant enzyme activities and osmolyte accumulation. RNA-seq analysis identified 1496 DEGs following pathogen challenge. Enrichment analysis indicated that soybean resistance is primarily mediated through the MAPK signaling pathway–plant, plant–pathogen interaction, and thiamine metabolism pathways. Integrated with qRT-PCR validation, *GmCML* was identified as a key gene conferring resistance against *F. oxysporum*. *GmCML* enhances root length and fresh root weight by elevating antioxidant enzyme activities and promoting the accumulation of osmoregulatory substances, thereby improving disease resistance. The variation in resistance among materials may largely be due to functional polymorphisms in the promoter region of *GmCML*, which also appears to have undergone positive selection during domestication. Our study on the *GmCML* gene opens multiple avenues for improving disease resistance in soybean germplasms.

## Data Availability

The clean reads were submitted to the sequence read archive of the National Center for Biotechnology Information (NCBI) under BioProject ID No: PRJNA1298383.

## Supplementary Materials

The following supporting information can be downloaded at https://www.mdpi.com/article/10.3390/plants14203222/s1, Table S1. Summary of RNA-seq raw data. Table S2. Quality control and QC analysis of RNA-seq data. Table S3. DEGs in key metabolic pathways enriched in KEGG analysis. Table S4. DEGs shared by MAPK signaling pathway (plant) and plant–pathogen interaction. Table S5. Primers used in this study.
